# Longitudinal study of age-specific pattern of coronavirus infection in Lyle’s flying fox (*Pteropus lylei*) in Thailand

**DOI:** 10.1186/s12985-018-0950-6

**Published:** 2018-02-20

**Authors:** Supaporn Wacharapluesadee, Prateep Duengkae, Aingorn Chaiyes, Thongchai Kaewpom, Apaporn Rodpan, Sangchai Yingsakmongkon, Sininat Petcharat, Patcharakiti Phengsakul, Pattarapol Maneeorn, Thiravat Hemachudha

**Affiliations:** 1 Thai Red Cross Emerging Infectious Diseases - Health Science Centre, World Health Organization Collaborating Centre for Research and Training on Viral Zoonoses, Chulalongkorn Hospital, Faculty of Medicine, Chulalongkorn University, Bangkok, Thailand; 2 Faculty of Forestry, Kasetsart University, Bangkok, Thailand; 3 Faculty of Veterinary Medicine, Kasetsart University, Bangkok, Thailand; 4 Department of National Parks, Wildlife and Plant Conservation, Bangkok, Thailand

**Keywords:** Coronavirus, Chiroptera, *Pteropus*, Thailand

## Abstract

**Background:**

Bats are natural reservoirs for several highly pathogenic and novel viruses including coronaviruses (CoVs) (mainly *Alphacoronavirus* and *Betacoronavirus*). Lyle’s flying fox (*Pteropus lylei*)‘s roosts and foraging sites are usually in the proximity to humans and animals. Knowledge about age-specific pattern of CoV infection in *P. lylei*, prevalence, and viral shedding at roosts and foraging sites may have an impact on infection-age-structure model to control CoV outbreak.

**Methods:**

*P. lylei* bats were captured monthly during January–December 2012 for detection of CoV at three areas in Chonburi province; two human dwellings, S1 and S2, where few fruit trees were located with an open pig farm, 0.6 km and 5.5 km away from the bat roost, S3. Nested RT-PCR of *RNA-dependent RNA polymerase* (*RdRp*) gene from rectal swabs was used for CoV detection. The strain of CoV was confirmed by sequencing and phylogenetic analysis.

**Results:**

CoV infection was found in both juveniles and adult bats between May and October (January, in adults only and April, in juveniles only). Of total rectal swab positives (68/367, 18.5%), ratio was higher in bats captured at S1 (11/44, 25.0%) and S2 (35/99, 35.4%) foraging sites than at roost (S3) (22/224, 9.8%). Juveniles (forearm length ≤ 136 mm) were found with more CoV infection than adults at all three sites; S1 (9/24, 37.5% vs 2/20, 10%), S2 (22/49, 44.9% vs 13/50, 26.0%), and S3 (10/30, 33.3% vs 12/194, 6.2%). The average BCI of CoV infected bats was significantly lower than uninfected bats. No gender difference related to infection was found at the sites. Phylogenetic analysis of conserved *RdRp* gene revealed that the detected CoVs belonged to group D *betacoronavirus* (*n* = 64) and *alphacoronavirus* (*n* = 4).

**Conclusions:**

The fact that CoV infection and shedding was found in more juvenile than adult bats may suggest transmission from mother during peripartum period. Whether viral reactivation during parturition period or stress is responsible in maintaining transmission in the bat colony needs to be explored.

## Background

Coronaviruses (CoVs) are classified into four genera: *Alphacoronavirus* (αCoV), *Betacoronavirus* (βCoV), *Gammacoronavirus*, and *Deltacoronavirus* [1]. CoVs infect wide variety of mammals and birds, causing upper and lower respiratory, hepatic, enteric and neurological illnesses with varying severity. Bat CoVs (BtCoVs) are likely the gene source of αCoV and βCoV, while avian CoVs are sources of *Gammacoronavirus*, and *Deltacoronavirus* [2]. Although there is single lineage in αCoV, βCoVs are further separated into four lineages (A – D) [3]. Lineage A βCoV, including bovine CoVs, human CoV (HCoV)-OC43 and related viruses, have been detected in various mammals such as cows, horses, deer, antelopes, camels, giraffes, waterbucks, dogs, and humans worldwide, but not in bats. Lineages B-D βCoVs have been detected in bats worldwide [4].

Currently, six CoV strains are known to cause human infection; four CoVs cause mild respiratory illness, including two αCoVs: HCoV-NL63 and HCoV-229E, and two βCoVs: HCoV-HKU1and HCoV-OC43 [5]. The other two βCoVs cause severe respiratory tract infection with high-fatality rates, such as severe acute respiratory syndrome (SARS) and Middle East respiratory syndrome (MERS), belonging to lineages B and C, respectively. Bat related MERS-CoVs phylogenetically-related to humans have been previously discovered in *Tylonycteris pachypus* (BtCoV-HKU4) and *Pipistrellus abramus* (BtCoV-HKU5) in Hong Kong, in 2006 [6], *Neoromicia capensis* (NeoCoV) from South Africa, in 2011 [7], and *Pipistrellus* cf. *hesperidus* (PREDICT/PDF-2180 CoV) from Uganda, in 2013 [8]. SARS-like BtCoV was initially identified from the genus *Rhinolophus* in 2005, after the SARS outbreak in humans in 2002–2003, and identification of virus in palm civets (*Paguma larvata*) from live animal market in Guangdong, China in 2003 [9, 10].

BtCoVs have been identified in many insectivorous and frugivorous (family Pteropodidae) bats on many continents: America, Europe, Africa, and Asia [4]. Different species of Pteropodidae have been identified as a major source of lineage D βCoV (HKU9) in Africa (*Rousettus aegyptiacus*, Kenya [11], *Pteropus rufus* and *Eidolon dupreanum*, Madagascar [12]), and Asia (*R. leschenaulti*, China [13], *Cynopterus brachyotis*, Philippines [14], *Ptenochirus jagori*, Philippines [15], *Pteropus giganteus*, Bangladesh [16], *Cynopterus sphinx*, Thailand [17].

Thailand is home to 146 bat species (125 insectivorous and 21 frugivorous) [18]. The prevalence and diversity of BtCoVs in Thailand has been studied in the last decade [17, 19, 20]. CoVs were found in 11 insectivorous bat species and in 2 frugivorous bat species. However, data from *Pteropus* bats have been lacking despite *Pteropus* being the biggest colony of Pteropodidae in Thailand. Three species *(P. lylei*, *P. vampyrus* and *P. hypomelanus*) are reservoirs of Nipah virus (NiV) in Thailand [21]. The prevalence of NiV RNA in urine of *P. lylei* has been seasonally detected during the months of May and June [22].

*P. lylei* (Lyle’s flying fox (LFF)) ranges from Yunnan in China, and extends to Cambodia, Thailand, and Vietnam [23]. Up to 20 colonies have been identified in Thailand [24] and the largest known colony comprises of about 10,000 individuals [22]. It shares foraging areas with other frugivorous bats in fruit trees, from which the fruits are also shared by humans. Moreover, trees in populated temple grounds and cultivated land are common roosting sites for LFF. Thus, consumption of partially eaten fruit, uncooked meat, or contact with saliva, urine or faeces, which can be contaminated with bat viruses, poses a risk of viral transmission from LFF to humans or domestic animals.

The potential for emergence of zoonotic viruses into the human population depends on the prevalence of the virus in its host species, host range mutations within viral quasispecies, and the degree to which the reservoir host interacts with humans [25]. To better understand the prevalence, persistence, phylogeny, and potential for interaction with humans, here we describe a comprehensive longitudinal study to detect CoV in LFF, and factors influencing infectivity. Bat rectal swabs were collected monthly from their roosting area and from two human dwellings (foraging sites) nearby. Individual bats were weighed and forearm (FA) lengths were measured for further characterization on its body composition index (BCI). Our results demonstrated for the first time that α- and β-CoVs are endemically circulating in LFFs in Thailand, and that age and BCI are significantly different between infected and uninfected bats.

## Methods

### Study sites

LFFs were captured from the largest colony in Thailand (total population of around 10,000 bats) [22] at Chonburi province (Luang temple, 13,830,018.9”N, 101809054.9″E, 6 m asl) in Central Thailand. Bats were sampled from three different sites: two human dwellings (bat foraging areas) situated at a mean distance of 0.6 km (S1) and 5.5 km (S2 with a small open-system pig farm, 40 pigs) from a bat roost, and the bat roost (S3). Sampling was carried out under protocols approved and permitted by the Department of National Parks, Wildlife and Plant Conservation, Thailand (No. 0909.204/2686) and the Animal Use Protocol No.1473001 approved by Chulalongkorn University Animal Care and Use Committee.

### Bat capture and sample collection

LFFs were captured monthly during January – December 2012 from the three sites, S1–3 (Table 1). At S1 and S2, bats were captured 2 nights per month, where the nets were set in the late evening, and captured animals were removed immediately. At S3, 10–20 bats were captured using mist-nets on the same nights as S1 and S2. Bats were not euthanized, and they were released after measurements were taken and samples were collected. Bats were identified morphometrically and species, sex, reproductive status, FA length and body mass were determined. Rectal swab was collected from each individual bat and immediately put into Lysis buffer (bioMérieux, SA, France). The samples were transported to laboratory on ice within 48 h and stored at -80^o^ C until further analysis.

**Table 1:** CoV positive bats, categorized by possible influential factors - collection site, age, sex and BCI (367 bats)

Possible influential factor		CoV PCR results: No. Positive / no. of tested (%)			
		S1 (house)	S2 (pig farm)	S3 (roost)	ALL SITES
Age^a^	Juvenile (J)	9/24 (37.5)	22/49 (44.9)	10/30 (33.3)	41/103 (39.8)
	Adult (A)	2/20 (10.0)	13/50 (26.0)	12/194 (6.2)	27/264 (10.2)
	Total	11/44 (25.0)	35/99 (35.4)	22/224 (9.8)	68/367 (18.5)
	Ratio (J:A)	24:20 = 1.20	49:50 = 0.98	30:194 = 0.15	103:264 = 0.39
Sex	Male	6/27 (22.2)	16/51 (31.4)	14/142 (9.9)	36/220 (16.4)
	Female	5/17 (29.4)	19/48 (39.6)	8/82 (9.8)	32/147 (21.8)
	Total	11/44 (25.0)	35/99 (35.4)	22/224 (9.8)	68/367 (18.5)
Mean BCI^b^ (352 bats)	All bats	1.83	1.94	2.90	2.50
	Positive	1.63	1.76	2.15	1.86
	Negative	1.89	2.04	2.99	2.65

^a^J = juvenile bat (forearm length ≤ 136 mm) A = adult bat (forearm length > 136 mm)

^b^Mean BCI is calculated from 352 bats those FA length and body mass were measured

### Nucleic acid extraction and CoV RNA detection

Total nucleic acid was extracted from 1 ml of suspended rectal swab using easyMAG® platform (bioMérieux, SA, France). Elution volume was 50 μl. Hemi-nested Reverse Transcription PCR (RT-PCR) was performed using broadly reactive consensus PCR primers for CoV, targeting the *RNA-dependent RNA polymerase* (*RdRp*) gene. A total of 5 μl of extracted nucleic acid was added to 50 μl of reaction mixture of OneStep RT-PCR kit (QIAGEN, Hilden, Germany), per manufacturer’s instructions, and reacted with each forward primer and reverse primer [14]. Hemi-nested PCR amplifications were performed using 2 μl of first amplification product and 48 μl of reaction mixture containing 1.0 unit of Platinum Taq DNA polymerase in 2.5 mM MgCl_2_, 400 μM dNTPs, 0.6 μM of second forward primer and 0.6 μM of the same reverse primer as the first round of RT-PCR. Amplification product of 434 bp was visualized using 2% agarose gel electrophoresis. All positive PCR products were further sequenced for confirmation and strain characterization.

### Sequencing and phylogenetic analysis

The *RdRp* PCR products were gel purified using the NucleoSpin® Gel and PCR Clean-up kit (MACHEREY-NAGEL GmbH & Co. KG), and sequenced directly using an automated ABI PRISM 377 DNA sequencer. When multi peaks were shown in chromatogram at same position from direct sequencing, PCR products were cloned using the pGEM®-T Easy Vector System and the LigaFast™ Rapid DNA Ligation System (Promega) before sequencing. Five colonies were picked up for sequencing. Sequences were cleaned using Bio-edit program and aligned with reference sequences collected from GenBank. Alignments were performed using Multiple Alignment using Fast Fourier Transform (MAFFT) [26]. Phylogenetic trees were created based on 357 and 299 bp *RdRp* gene sequence using the maximum likelihood method. Bootstrap values were determined using 1000 replicates via RaxmlGUI 1.3 with outgroup (Bulbul CoV/HKU11–934/*Pycnonotus jocosus*/CHN/2007/FJ376619) using the GTRI substitution model [27]. The phylogenetic tree was visualised using the FigTree program, version 1.4.2 [28].

### Statistical analysis

We considered the relative level of CoV infection in variables of bat. We used Chi-square and Fisher’s exact tests to determine the prevalence pattern of CoV by examining whether cues recorded in each kind of variables (location, sex, age and season) differed from expected. All statistical tests were completed in R statistic computing (version 3.2.2) with *p* < 0.05 interpreted as being statistically significant. The body condition index (BCI) was defined as body mass divided by FA length. To assess differences in BCI between CoV infected and uninfected bats, ANOVA with Tukey’s test for pair-wise comparisons was used for analysis.

## Results

### Sample collection

Only the *P. lylei* species (LFF) was included in this study. A total of 367 bats (220 male and 147 female) were captured and sampled. Total number of captured bats from sites S1, S2 and S3 were 44, 99, and 224 respectively (Table 1). FA length (**≤** 136 mm) was used to distinguish between juvenile and adult [29]. Body mass and FA lengths were determined for 352 bats (95.9%). FA lengths of juveniles (*n* = 96) ranged from 79.23–136.0 mm, and in adults (*n* = 256) ranged from 136.47–170.0 mm. Body mass of juveniles ranged from 124.0–307.0 g, while adults ranged from 212.0–658.0 g. The BCI in juveniles and adults ranged from 1.08–2.32 and 1.43–4.27 respectively. The ratio of juvenile and pup per adult bats captured from sites S1 (24:20, 1.2) and S2 (49:50, 0.9) were similar, but a lower ratio was found at site S3 (30:194, 0.15) (Table 1). Number of bats trapped/captured each month varied between 14 and 46 bats; minimum of 10 was captured each month at their roost (S3) as control. Juvenile bats were not captured in January through to March for testing, as the ratio of juvenile and adult bats in natural population is low due to the LFF’s breeding cycle, which is once a year from November to February [22]. Thus new-borns are delivered in February/March, and weaning juvenile bats are mostly observed in May. Of the 147 females captured, 18 were at active breeding age evident by either lactating (*n* = 10, which the specimens were available for testing from 9 pups), being pregnant (*n* = 4) or having enlarged nipples indicative of previous lactation (*n* = 4).

### Virus detection-prevalence

Sixty eight of 367 (18.5%) rectal swabs from LFF were positive for CoV by family wide CoV PCR [14]. As shown in Table 1, CoV RNA positive bats were found in 16.4% (36/220) male bats, and 21.8% (32/147) in female bats. There was no significant difference in the rate of infectivity when comparing sex (*p* > 0.05).

The number of CoV positive juvenile and adult bats from all three sites (*n* = 367 bats) were 39.8% (41/103) and 10.2% (27/264), respectively (Table 2). CoV positive juvenile bats from sites S1-S3 were 37.5% (9/24), 44.9% (22/49), and 33.3% (10/30), respectively. CoV positive adult bats from sites S1-S3 were 10% (2/20), 26.0% (13/50), and 6.2% (12/194), respectively (Table 1). Statistical analysis showed that CoV infectivity between juvenile and adult bats significantly differed (*p* < 0.01), indicating that CoV infection favoured juvenile LFFs.

**Table 2:** Number of bats PCR-positive for coronavirus by month and age^a^ from SI, S2 and S3

Month	Juvenile		Adult		Total	
	Tested	Positive (%)	Tested	Positive (%)	Tested	Positive (%)
January	0	0 (0)	14	4 (28.6)	14	4 (28.6)
February	0	0 (0)	14	0 (0)	14	0 (0)
March	0	0 (0)	20	0 (0)	20	0 (0)
April	12	2 (16.7)	34	0 (0)	46	2 (4.3)
May	21	13 (61.9)	18	3 (16.7)	39	16 (41.0)
June	20	11 (55. 0)	9	3 (33.3)	29	14 (48.3)
July	21	7 (33.3)	25	7 (28.0)	46	14 (30.4)
August	9	4 (44.4)	26	5 (19.2)	35	9 (25.7)
September	12	3 (25.0)	23	3 (13.0)	35	6 (17.1)
October	4	1 (25.0)	23	2 (8.7)	27	3 (11.1)
November	3	0 (0)	33	0 (0)	36	0 (0)
December	1	0 (0)	25	0 (0)	26	0 (0)
Total	103	41 (39.8)	264	27 (10.2)	367	68 (18.5)

^a^Juvenile bat: forearm length ≤ 136 mm

None of the rectal swabs from the 18 pregnant or lactating adult female bats tested positive for CoV. Interestingly, three attached pubs, but not their mothers, from a total of 9 pairs were found positive for CoV RNA. BCI of the two pups with available data were 1.69 (211 g body mass/125 mm FA) and 1.42 (170 g body mass/120 mm FA), which were lower than the uninfected mean for juvenile bats (1.72) (Table 3).

**Table 3:** Range and mean of forearm (FA), body mass, and body condition index (BCI) of bats in this study (352 bats) classified by age and/or CoV infection status

Bat characteristics	FA (mm) Range (mean)	Body Mass (g) Range (mean)	BCI Range (mean)
CoV positive bats	117–160.28 (135.14)	141–538 (255.19)	1.11–3.36 (1.86)
CoV negative bats	79.23–170.0 (147.55)	124–658 (396.47)	1.08–4.27 (2.65)
CoV positive juvenile bats	117–136 (128.25)	141–295 (206.75)	1.11–2.26 (1.61)
CoV negative juvenile bats	79.23–135.82 (128.82)	124–307 (221.79)	1.08–2.32 (1.72)
CoV positive adult bats	136.67–160.28 (145.34)	212–538 (326.96)	1.52–3.36 (2.23)
CoV negative adult bats	136.47–170 (151.51)	215–658 (439.19)	1.43–4.27 (2.88)
Total Juvenile bats	79.23–136.0 (128.58)	124–307 (215.52)	1.08–2.32 (1.67)
Total Adult bats	136.47–170 (150.86)	212–658 (427.35)	1.43–4.27 (2.81)

The prevalence of CoV infection in bats from sites S1-S3 were 25.0% (11/44), 35.4% (35/99) and 9.8% (22/224), respectively (Table 1). Statistical analysis showed that the number of CoV infected bats at the different sites differed significantly (*p* < 0.01). Frequency of viral detection was higher at these two foraging sites than the roost (χ^2^ = 36.31, *p* < 0.001). However, the prevalence of CoV infection in juvenile bats from the 3 sites was similar, 37.5%, 44.9%, and 33.3%, respectively (Table 1). Age and conditions of bats which may reflect their physical health and fitness may influence the selection of foraging site and their vulnerability to infection. During the same year of study, tracked bats from this colony mostly foraged in farmland, plantations, and gardens with the maximum linear distances from 2.2–23.6 km between day roosts and foraging areas [30].

### Temporal dynamics of viral shedding

Combining data from all sites, the CoV positive bats were found in 8 of 12 months, except February, March, November and December. High prevalent seasons were from May to August, with highest in June (14/29, 48.3%). Highest prevalence in juveniles were found in May (13/21, 61.9%), and in adults in June (3/9, 33.3%). There was higher prevalence of CoV infection among juvenile than adult bats during April–October (Table 2). In January, 4 CoV positive adult bats were found at S3. Individual BCI of the one female and 3 male bats were 2.83, 1.77, 1.84, 2.75, respectively, which were lower than the mean BCI in uninfected adult bats (2.88) (Table 3). We analysed the monthly prevalence of CoV infectivity in juvenile and adult bats, and combined (Table 2). There was significant difference in the seasonal prevalence of CoV infection and shedding in adults (*p* < 0.05), but not in juvenile bats or combined.

### BCI – Infected bats

**Fig. 1:**
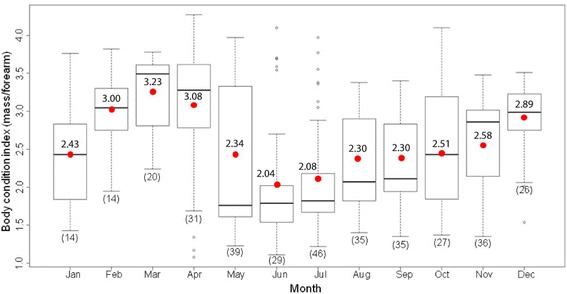
Body condition indices (BCI) of 352 bats captured in the study from January to December 2012. Bats were captured monthly at three sites (S1-S3). Numbers in brackets indicate sample size from 3 sites. Boxes depict the 25th and 75th percentiles, lines within boxes mark the median, red spot and number represent mean, whiskers represents minimum and maximum values, and circles indicates outliers

**Fig. 2:**
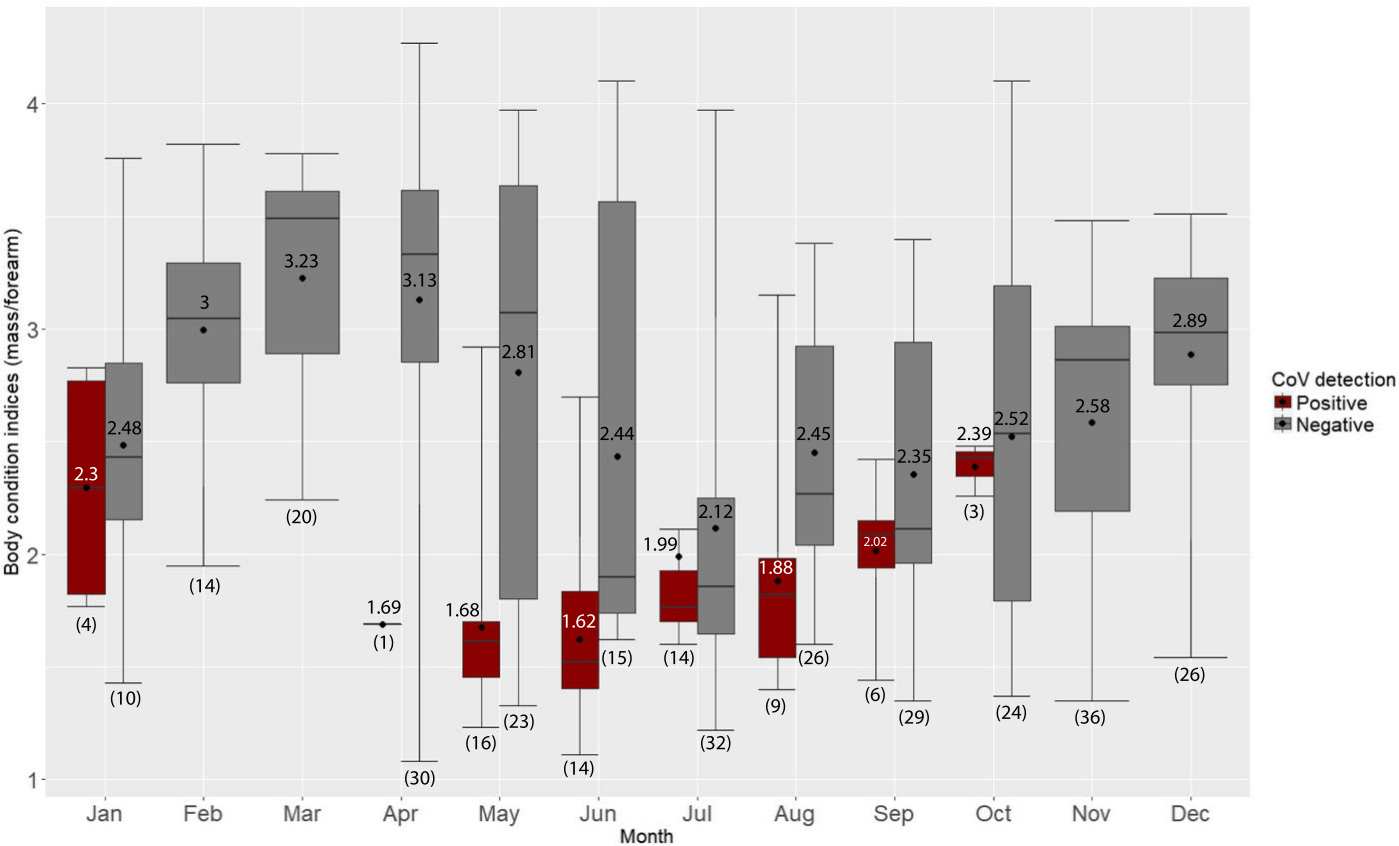
Body condition indices (BCI) of bats tested negative (gray) and positive (brown) in the study. Bats were captured monthly from January to December 2012 at three sites. Rectal swabs from 352 bats were tested for CoV by PCR. Numbers in brackets indicate sample size from 3 sites. Boxes depict the 25th and 75th percentiles, lines within boxes mark the median, spot and number represent mean, whiskers represents minimum and maximum values, and circles indicates outliers

**Fig. 3:**
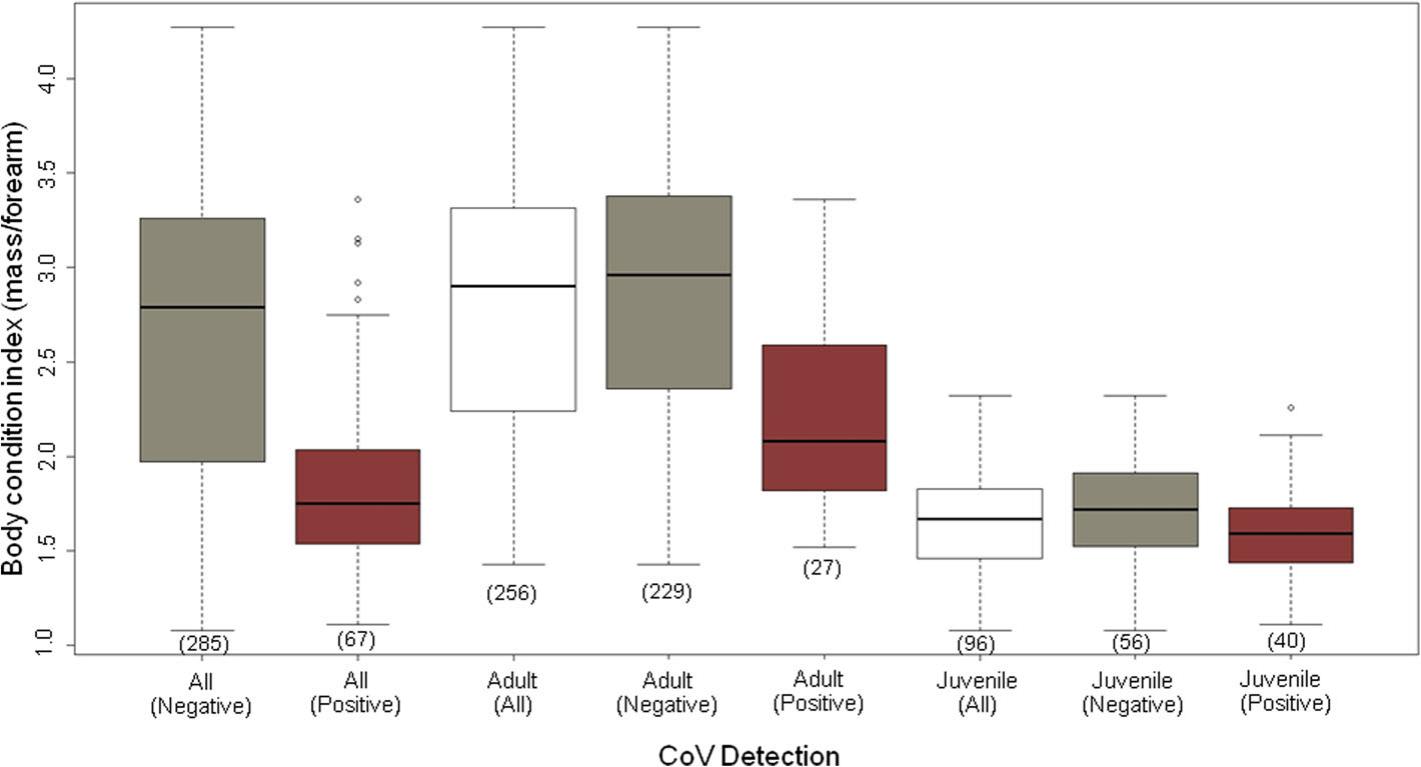
Body condition indices (BCI) of bats tested negative (gray) and positive (brown) in the study. Rectal swabs from 352 bats were tested for CoV by PCR. Forearm length **≤** 136 mm was used to classify bats as juvenile. Numbers in brackets indicate sample size. Boxes depict the 25th and 75th percentiles, lines within boxes marks the median, whiskers represents minimum and maximum values, and circles indicates outliers

There was significant difference between FA and body mass of CoV positive bats compared to uninfected bats (*p* value < 0.01). BCI analysis was performed on bats with complete data of FA and body mass (352 bats). The mean BCI of captured bats in the study varied each month (Fig. 1). BCI of total tested bats (Fig. 1) and uninfected bats (Fig. 2) show similar seasonality. The lowest mean BCI of both total number of tested bats and CoV-positive bats were found in June (mean 2.05 and 1.62 respectively) (Figs. 1 and 2) when CoV infection (48.28%) was most prevalent (Table 2). In uninfected bats, the lowest mean BCI was found in July (2.12) rather than June (2.44) (Fig. 2). From this study, the CoV infected bats had significantly lower mean BCI than uninfected bats, 1.86 and 2.65 respectively (*p* value < 0.01) (Table 3). The BCI of CoV infected bats varied between 1.11 and 3.36 (mean 1.86) whilst the BCI of uninfected bats was between 1.08 and 4.27 (mean 2.65). The BCI of uninfected juvenile bats was between 1.08 and 2.32 (mean 1.72), whilst the BCI of uninfected adult bats varied between 1.43 and 4.27 (mean 2.88). The BCI of CoV infected juvenile bats was between 1.11 and 2.26 (mean 1.61), whilst the BCI of CoV infected adult bats varied between 1.52 and 3.36 (mean 2.23). The mean BCI of infected juvenile and adult bats were significantly lower than uninfected juvenile and adult bats respectively, (*p* value < 0.01, *p* value < 0.01 respectively) (Fig. 3). There was statistically significant difference in the mean body mass (*p* value < 0.01) and mean FA length (*p* value < 0.01) between CoV infected and uninfected bats (Table 3).

### Phylogenetic analyses

**Fig. 4:**
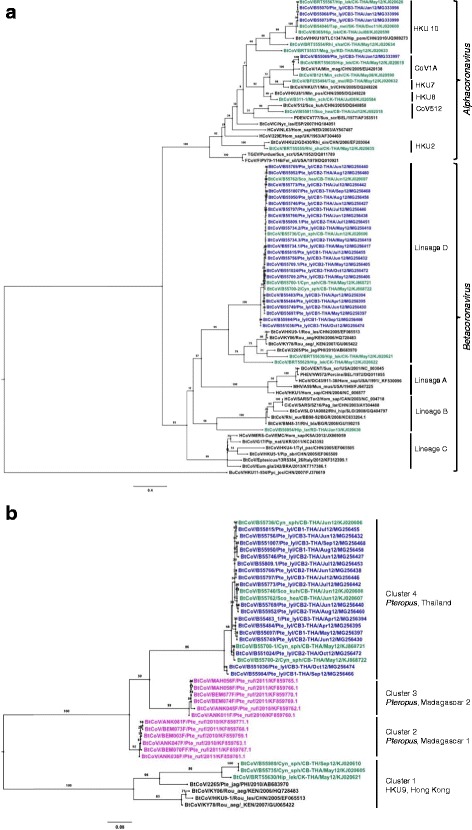
Maximum likelihood phylogenetic trees of coronavirus (CoV) generated using 357 (**a**-Bat CoVs) and 299 (**b**-Lineage D βCoVs) nucleotides of the *RdRp* gene sequences of CoVs from Thailand; in LFF (this study-blue), other bat species from previous study in Thailand [15] (green), from Madagascar’s *Pteropus rufus* [17] (**b**, pink) and reference strains of CoVs group (black). Only the representative sequences of LFF βCoVs were used for analysis. The raxmlGUI 1.3 and the GTRI substitution model with 1000 bootstrap were used for generating both phylogenetic trees. Trees were visualized using FigTree 1.4.2. BuCoV/HKU11–934/Pyc_joc/CHN/2007/FJ376619 was used as an outgroup for tree A

Sixty-eight CoV sequences were deposited in GenBank with accession MG256395-MG256474 and MG333996-MG333999. Phylogenetic analysis of 357 bp of *RdRp* gene using raxmlGUI program revealed that 64 of 68 detected CoVs belonged to βCoV genus, roosting with Hong Kong strain, BtCoV HKU9 (*R. lechenaulti*, EF065513) and Kenya (*R. aegyptiacus*, GU065422), while the other 4 belonged to group 1A αCoV (Fig. 4a). The βCoVs from this study clustered in the same clade and shared 95.5–100% nucleotide identity with each other (98.3–100% identity of 118 amino acids). Two individual bats (BRT55709 and BRT55734) were found to be co-infected with multiple strains of the same βCoV species (difference of 1–2 amino acids). These viruses had amino acids differing from the HKU9 BtCoV, group D βCoV reference strain by 11.7–14.2%. They formed a different clade to other CoVs from the same bat genus (*Pteropus*) from Madagascar’s *P. rufus* (Fig. 4b). However, they were in the same clade with CoVs from different bat species captured at the same site with this study; *Cynopterus sphinx, Scotophilus heathii,* and *Scotophilus kuhlii* (Genbank accession numbers KJ868722, KJ020607, KJ020608, respectively) [17].

## Discussion

This is the first longitudinal study of CoV infection in wild bats in Thailand, where 367 LFF bats were captured monthly for one year at one roosting site and two foraging sites close to the bat roost. One fourth of bats were juvenile, and 59.9% were male.

The ratios between captured juvenile and adult bats were different at the bat roost and foraging sites. Only 13% of juvenile (30/224) bats were captured at the bat roost in the year of the study, whereas half of the juvenile bats were captured from both foraging sites (24/44, 49/99, from S1 and S2 respectively). The maximum linear distances between roosts and foraging areas of LFF at this site varied from 2.2–22.3 km [30]. Foraging sites near roost, even with limited food sources, may be practical for young or unhealthy bats that are unable to fly far.

CoV RNA was detected in approximately 18% of all bats sampled, which is in the same range as the study in China (16%, [31]; 15.8%, [32]), and Germany (9.8%, [33]). The prevalence of CoV infection in *Pteropus* bats (*P. rufus*) from Madagascar was similar to this study (17.1%, 13/76) [12]. On the other hand, the prevalence in this study was higher than the two previous studies in Thailand by Wacharapluesadee et al. (6.7%, 47/626) [17] and Gouilh et al. (10.5%, 28/265) [20]. This may be the result of a bias from the cross sectional study of these two previous studies or an indication of difference in prevalence rate in different bat species.

Ratios of captured bat genders in this study were roughly similar at foraging sites. At the roost, male bats were predominantly captured. CoV infection was not correlated with sex of bat, neither at the roost nor at the foraging sites. This finding is similar to the studies from Germany [33] and Colorado, USA [25].

In our study, CoV infection was found to be associated with younger ages; 39.8% of juvenile bats versus 10.2% adult bats were positive for CoV RNA. Similar findings have been reported from the study in insectivorous bats from USA (19% juvenile versus 9% adult bats positive for CoV) [25] and Vespertilionid bats in Germany (23.7% juvenile versus 15.9% sub-adult versus 8.5% adult bats positive for CoV) [33]. These findings support the hypothesis that young bats may be more susceptible to CoV infection, and serves to propagate and play an important role in maintaining the virus within bat colonies. The divergence in rate of CoV infection from different study sites (Table 1) was likely to be influenced by the age and body condition of bats.

Three of 9 unweaned pups were CoV RNA positive, while their mothers and all lactating female carrying pups were negative for CoV. It may be possible there was a placental transmission, after which the virus was then cleared from adult female bats. Another possibility is that the unweaned bats acquired infection from contaminated secretion of other bats hanging from the same tree. However, the study by Gloza-Rausch et al. 2008 [33], where 54 of 178 (30%) of studied female bats were lactating, found higher rate of CoV infection in lactating bats (22.4%) than in non-lactating bats (9.7%) which supports the first scenario. It is to be noted that limited number of lactating bats were included in our study (9 of 147, 6.1%). Targeting mother-pup pairs in future studies would be required to confirm the vertical (placental) transmission of CoV in LFF.

Seasonal prevalence was mostly related to the number of juvenile bats captured for testing in each month (Table 2), except in January when all four CoV positive bats were adult. Notably, these positive adult bats had lower BCI (2.83, 1.77, 1.84, 2.75) than the mean uninfected adult bats (2.88). Three of the 4 infected adult bats had lower body mass (444, 429, 258, 276 g) than mean uninfected adult bats (439 g). The mean body mass of infected bats was significantly lower than in uninfected bats (Table 3). This is similar to the study where *Hipposideros pomona* bats in Hong Kong with HKU10 CoV infection had lower body mass than uninfected bats, even though they appeared to be healthy [34]. These bats seemed to be in poor condition, serving as the other group in addition to juvenile bats that further maintained the virus within the population.

Sixty eight CoVs were detected from this study, forming 2 genetically distinct strains. Sixty four belonged to βCoV (SARS-related group) with relatively close homology to the reference virus, BtCoV-HKU9 [6]. Four belonged to αCoV, and their sequences related to CoVs previously detected in insectivorous bats in Thailand such as *H. lekaguli*, *H. armiger* and *Taphozous melanopogon* [17]. This supports the possibility of interspecies transmission, rather than virus-host specific sharing, between bats of different suborder (*Pteropus* in Pteropodidae, Hipposideridae and Emballonuridae) that do not share food, foraging sites, or roosts, similar to the earlier HKU10 CoV study between *R. leschenaulti* and *H. pomona* bats [15]. The evolution of CoVs in different host species-order should be further studied in order to understand the route of spillover and transmission.

Bats from different species-genus that share foraging sites may also share infections and particular CoV strains, for example βCoV from LFF (this study), *C. sphinx*, *S. Heathii,* and *S. kuhlii* [17] (Fig. 4a-b). βCoV from same bat genus in different geographic region displayed distinct clusters (Fig. 4b), for example *P. rufus* from Madagascar (cluster 2–3) [12] and LFF from this study (cluster 4). This demonstrated the βCoV inclines interspecies sharing rather than virus-host specific sharing.

Given the mobility of LFF in Thailand, where the maximum linear distance between day roosts and foraging areas for LFF is 23.6 km [29], and its tendency for sharing habitat with other colonies, the detected strains of CoVs from this study may be found in LFFs all over the region. The high prevalence of CoV in this study suggests circulation of infection within the bat colony. Study of CoV diversity from other LFF colony in Thailand and region is required to improve our understanding of the evolution and spillover patterns of CoV.

## Conclusions

Our study found that CoV transmission in LFF occurred throughout the year at a baseline level, and the months surrounding the birthing season (May–August) represented times of increased infection among juveniles. The CoV prevalence in LFF related mostly to the age of bat rather than location, sex or season. The interspecies transmission of CoV among different bat genus or family demonstrated the possibility of spillover and the potential for emergence of zoonotic viruses into the human population. This data provides the first long-term monitoring of CoV circulation in nature and identifies ecological drivers. The relationship between animal age and infectivity to other bat species should be further investigated to confirm this phenomenon. Additional studies on CoV diversity among *Pteropus* bat species in Thailand and neighbouring countries, as well as aspects of the virus-host interaction are needed to understand the origins, evolution, maintenance patterns, dispersal and zoonotic potential of CoV across the region.

## Abbreviations

ANOVA: Analysis of variance
BCI: Body composition index
bp: Base pairs
BtCoV: Bat coronavirus
CoV: Coronavirus
DNA: Deoxyribonucleic acid
FA: Forearm
LFF: Lyle’s flying fox
MERS: Middle East respiratory syndrome
NiV: Nipah virus
nt: nucleotides
PCR: Polymerase Chain Reaction
*RdRp*: RNA-dependent RNA polymerase
RNA: ribonucleic acid
RT-PCR: Reverse Transcription - Polymerase Chain Reaction
S1: Study site 1 – human dwelling (0.6 km from bat roost)
S2: Study site 2 – human dwelling, small open-pig farm (40 pigs) (5.5 km from bat roost)
S3: Study site 3 – Bat roost
SARS: Severe acute respiratory syndrome
αCoV: *Alphacoronavirus*
βCoV: *Betacoronavirus*
